# The Prevalence of Sexual Behavior Stigma Affecting Gay Men and Other Men Who Have Sex with Men Across Sub-Saharan Africa and in the United States

**DOI:** 10.2196/publichealth.5824

**Published:** 2016-07-26

**Authors:** Shauna Stahlman, Travis Howard Sanchez, Patrick Sean Sullivan, Sosthenes Ketende, Carrie Lyons, Manhattan E Charurat, Fatou Maria Drame, Daouda Diouf, Rebecca Ezouatchi, Seni Kouanda, Simplice Anato, Tampose Mothopeng, Zandile Mnisi, Stefan David Baral

**Affiliations:** ^1^ Center for Public Health and Human Rights Department of Epidemiology Johns Hopkins University Baltimore, MD United States; ^2^ Department of Epidemiology Rollins School of Public Health Emory University Atlanta, GA United States; ^3^ Division of Epidemiology and Prevention Institute of Human Virology University of Maryland Baltimore, MD United States; ^4^ Enda Santé Dakar Senegal; ^5^ Department of Geography School of Social Sciences Gaston Berger University Saint-Louis Senegal; ^6^ Enda Santé Abidjan Côte d'Ivoire; ^7^ Institut de Recherche en Sciences de la Santé-IRSS Ouagadougou Burkina Faso; ^8^ Institut Africain de Santé Publique Ouagadougou Burkina Faso; ^9^ Arc-en-ciel Lomé Togo; ^10^ Matrix Support Group Maseru Lesotho; ^11^ Ministry of Health Mbabane Swaziland

**Keywords:** stigmatization, social stigma, HIV, male homosexuality, United States, Western Africa, Southern Africa, mental health

## Abstract

**Background:**

There has been increased attention for the need to reduce stigma related to sexual behaviors among gay men and other men who have sex with men (MSM) as part of comprehensive human immunodeficiency virus (HIV) prevention and treatment programming. However, most studies focused on measuring and mitigating stigma have been in high-income settings, challenging the ability to characterize the transferability of these findings because of lack of consistent metrics across settings.

**Objective:**

The objective of these analyses is to describe the prevalence of sexual behavior stigma in the United States, and to compare the prevalence of sexual behavior stigma between MSM in Southern and Western Africa and in the United States using consistent metrics.

**Methods:**

The same 13 sexual behavior stigma items were administered in face-to-face interviews to 4285 MSM recruited in multiple studies from 2013 to 2016 from 7 Sub-Saharan African countries and to 2590 MSM from the 2015 American Men’s Internet Survey (AMIS), an anonymous Web-based behavioral survey. We limited the study sample to men who reported anal sex with a man at least once in the past 12 months and men who were aged 18 years and older. Unadjusted and adjusted prevalence ratios were used to compare the prevalence of stigma between groups.

**Results:**

Within the United States, prevalence of sexual behavior stigma did not vary substantially by race/ethnicity or geographic region except in a few instances. Feeling afraid to seek health care, avoiding health care, feeling like police refused to protect, being blackmailed, and being raped were more commonly reported in rural versus urban settings in the United States (*P*<.05 for all). In the United States, West Africa, and Southern Africa, MSM reported verbal harassment as the most common form of stigma. Disclosure of same-sex practices to family members increased prevalence of reported stigma from family members within all geographic settings (*P*<.001 for all). After adjusting for potential confounders and nesting of participants within countries, AMIS-2015 participants reported a higher prevalence of family exclusion (*P*=.02) and poor health care treatment (*P*=.009) as compared with participants in West Africa. However, participants in both West Africa (*P*<.001) and Southern Africa (*P*<.001) reported a higher prevalence of blackmail. The prevalence of all other types of stigma was not found to be statistically significantly different across settings.

**Conclusions:**

The prevalence of sexual behavior stigma among MSM in the United States appears to have a high absolute burden and similar pattern as the same forms of stigma reported by MSM in Sub-Saharan Africa, although results may be influenced by differences in sampling methodology across regions. The disproportionate burden of HIV is consistent among MSM across Sub-Saharan Africa and the United States, suggesting the need in all contexts for stigma mitigation interventions to optimize existing evidence-based and human-rights affirming HIV prevention and treatment interventions.

## Introduction

The World Health Organization, UNAIDS, and the White House National HIV/AIDS Strategy have collectively called for the need to reduce stigma toward key populations to reduce the impact of human immunodeficiency virus (HIV) and to optimize HIV treatment outcomes [1-3]. In this context, gay men and other men who have sex with men (MSM) continue to be among the populations at highest risk for HIV infection worldwide [4,5]. The Centers for Disease Control and Prevention recently estimated that if current incidence rates continue, 1 in 6 MSM in the United States will be diagnosed with HIV in their lifetime, including 1 in 2 Black MSM, 1 in 4 Latino MSM, and 1 in 11 White MSM [6]. In low- and middle-income countries, MSM are estimated to have at least 19 times the odds of living with HIV compared with other reproductive-aged adults [7]. And in Sub-Saharan Africa, even in regions commonly characterized as generalized epidemics, the prevalence and incidence of HIV among MSM is consistently higher than that of age-matched men in all settings [8,9].

Sexual behavior stigma, defined here as stigma that is anticipated, perceived, or experienced as a result of one’s sexual experience [10], has been linked with adverse HIV-related outcomes among MSM including reduced rates of HIV testing and increased sexual risk practices leading to enhanced risk for HIV infection [11-14]. Separately, sexual behavior stigma has been linked to adverse mental health outcomes such as depression, suicidal ideation, and substance use disorders [15-17]. This stigma can operate at community- or structural-levels to negatively influence health outcomes. At the community level, sexual behavior–related stigma can limit the provision and uptake of sexual health services [18,19]. For example, culturally insensitive health workers may result in MSM avoiding HIV prevention services; or even more problematically, MSM living with HIV may avoid HIV treatment. Reduced use of health and HIV services by MSM, due to enacted or perceived discrimination, may limit knowledge of the risks of unprotected anal intercourse and opportunities for access to prevention services [20,21]. At the structural level, laws against same-sex practices can increase fear and avoidance of health care services, risk behaviors, stress, and promote violence, which can worsen health conditions for MSM and the broader communities [20,22-25].

Within the United States, HIV disproportionately affects young, Black and Hispanic MSM [26-32]. However, individual behavioral risk factors do not appear to explain the increased risk for HIV among racial/ethnic minorities [30,33]. Instead, the burden of stigma and mental health secondary to sexual and gender minority stress may contribute to the disparities observed around the world [34,35]. As a result, there is a growing recognition for the need to be able to measure and evaluate stigma toward MSM.

However, a consensus is lacking for how to quantify, measure, and evaluate sexual behavior stigma for key populations. Previous studies have identified high levels of sexual behavior stigma among MSM both in Sub-Saharan Africa and in the United States including physical assault and verbal harassment [23,36-39]. However, comparisons are imperfect because many studies do not use consistent metrics for measuring this stigma across settings [40]. Thus, the objectives of this paper are (1) to describe the prevalence of these sexual behavior stigma items in the United States and across age, racial/ethnic, population density, and regional subgroups, and (2) to make a comparison of sexual behavior stigma between MSM in 3 different global regions (United States, West Africa, and Southern Africa) using consistent metrics.

## Methods

### Study Population and Key Measures

#### American Men’s Internet Survey (AMIS)

The American Men’s Internet Survey (AMIS) is an annual cross-sectional behavioral survey of MSM in the United States [41,42]. AMIS-2015 recruited MSM from a variety of websites using banner advertisements or email blasts. Websites included general social networking sites (eg, Facebook), general gay interest sites (eg, sites that post news and articles relevant to a gay audience), gay social networking sites, and mobile-only geospatial social networking applications that connect men with other men based on their proximity. Approximately one-half of the surveys were performed on desktop computers and one-half on mobile phones. We used an interim survey dataset from September 2015 to March 2016. To be eligible to participate, men had to be aged 15 years or older, identify as the male gender, and report that they had oral or anal sex with a man at least once in the past. For the purpose of making comparisons with the African data sets, we limited the study sample to men who reported anal sex with a man at least once in the past 12 months and men who were aged 18 years and older, because these were eligibility requirements in the African studies. We also limited the AMIS-2015 sample to men with a current US residency as measured using reported zip codes and Internet protocol addresses. No incentive was provided to the participants.

AMIS consisted of a core questionnaire that was administered to all participants and included questions about demographics and disclosure status, which refers to whether the participant disclosed his same sex practices to either health care workers or family members. It also consisted of 3 different subset questionnaires to which participants were randomized at the start of the survey. All AMIS-2015 participants were asked if they identified as Hispanic/Latino, American Indian or Alaskan Native, Asian, Black or African American, Native Hawaiian or Pacific Islander, or White, and could choose all groups that apply. For the purpose of these analyses, we created a single variable for race/ethnicity, which grouped participants into the following mutually exclusive categories: Hispanic, non-Hispanic Black (“Black”), non-Hispanic White (“White”), and non-Hispanic Other (“Other”), which included those who identified as multiracial, Asian, and Native American/Hawaiian. Participants who indicated that they preferred not to answer questions about race, or that they didn’t know/did not apply were treated as missing (39/2551, 1.5%). We used a combination of county and zip code of residence to determine population density. Population density was classified as urban if participants were recruited from a county or zip code that had a population density of at least 1000 people per square mile, and rural if less than 1000 people per square mile. Sexual behavior stigma items were included in a subset questionnaire; therefore, one-third of participants were asked about sexual behavior stigma (2590/7853, 33.0%).

### Sub-Saharan Africa

Data from Sub-Saharan Africa have been presented in previous studies [23,43-45]. In this secondary analysis, we created a combined data set that was limited to MSM who were aged 18 years or older, reported being assigned male sex at birth and also identified as male, and reported anal intercourse with a man in the past 12 months. Data were collected predominantly via respondent driven sampling (RDS) [46] and snowball sampling [47] from 4285 MSM from 7 Sub-Saharan African countries (Table 1). RDS seeds were recruited from local MSM-affiliated community-based organizations (CBOs) although not all seeds were CBO members. Seeds were selected to represent the diversity of the target population with respect to demographics, HIV status, partnering, and other factors. There is significant consistency of methods across settings with face-to-face administration of a structured survey instrument by MSM or MSM-friendly staff members at the local CBOs. In addition, all data sets contain measures of sociodemographics, disclosure status, and sexual behavior stigma. For these analyses, population density was similarly classified as urban if participants were recruited from a study site located in a city that had a population density of at least 1000 people per square mile, and rural if less than 1000 people per square mile. Participants received a modest reimbursement for their time, the cost of travel to the study site (US$2-$6), and for each eligible participant they recruited into the study (for RDS studies).

**Table 1:** Summary of Sub-Saharan Africa MSM data sets.

Region	Country	Laws pertaining to same sex practices [48-50]	Recruitment dates	Study site locations	Recruitment method	Sample size
West Africa	Burkina Faso	Not criminalized	January-August 2013	Bobo Dioulasso and Ouagadougou	RDS^a^	443
West Africa	Cote d’Ivoire	Not criminalized	March-October 2015	Abidjan, Gagnoa, Bouaké, and Yamoussoukro	RDS	794
Southern Africa	Lesotho	Sodomy prohibited as common-law offence	February- September 2014	Maseru and Maputsoe	RDS	487
West Africa	Nigeria	Imprisonment	March 2013- August 2015	Abuja and Lagos	RDS	1067
West Africa	Senegal	Imprisonment	February- November 2015	Dakar, Mbour, and Thies	RDS	522
Southern Africa	Swaziland	Unenforced penalties	October- December 2014	Multiple	Snowball	419
West Africa	Togo	Imprisonment	January-June 2013	Kara and Lome	RDS	553

^a^Abb: respondent driven sampling.

### Sexual Behavior Stigma

Sexual behavior stigma was measured using the same 13 items in AMIS-2015 and in each of the Sub-Saharan Africa studies (Textbox 1). These measures include perceived, anticipated, and experienced stigma, such as stigma from family and friends, stigma from health care workers, and stigma from broader society. For questions pertaining to physical attacks and rape, we included responses as a “yes” only if the participant believed that these experiences were related to the fact that he has sex with men.

**Sexual behavior stigma items (response options: yes/no).**

1. Have you ever felt excluded from family activities because you have sex with men?

2. Have you ever felt that family members have made discriminatory remarks or gossiped about you because you have sex with men?

3. Have you ever felt rejected by your friends because you have sex with men?

4. Have you ever felt afraid to go to health care services because you worry someone may learn you have sex with men?

5. Have you ever avoided going to health care services because you worry someone may learn you have sex with men?

6. Have you ever felt that you were not treated well in a health center because someone knew that you have sex with men?

7. Have you ever heard health care providers gossiping about you (talking about you) because you have sex with men?

8. Have you ever felt that the police refused to protect you because you have sex with men?

9. Have you ever felt scared to be in public places because you have sex with men?

10. Have you ever been verbally harassed and felt it was because you have sex with men?

11. Have you ever been blackmailed by someone because you have sex with men?

12a. Has someone ever physically hurt you (pushed, shoved, slapped, hit, kicked, choked or otherwise physically hurt you)?

12b. Do you believe any of these experiences of physical violence was/were related to the fact that you have sex with men?

13a. Have you ever been forced to have sex when you did not want to? (By forced, I mean physically forced, coerced to have sex, or penetrated with an object, when you did not want to).

13b. Do you believe any of these experiences of sexual violence were related to the fact that you have sex with men?

### Ethics

AMIS-2015 data collection was approved by the human subjects research review board at Emory University. Sub-Saharan African studies were approved by respective in-country ethics committees: the Health Research Ethics Committee of Burkina Faso, the Health Research Ethics Committee of Côte d’Ivoire, the Lesotho National Health Research Ethics Committee, the Ethical Committee of Togo, the Senegalese National Health Research Ethics, the Swaziland Scientific Ethics Committee, and the institutional review board (IRB) at the Johns Hopkins Bloomberg School of Public Health. For Nigeria, approval was obtained by the Federal Capital Territory Health Research Ethics Committee, the University of Maryland Baltimore IRB, and the Walter Reed Army Institute of Research IRB. Informed consent was obtained from all individual participants included in these analyses.

### Statistical Analysis

We used descriptive statistics (frequencies, percentages) to describe the distribution of sexual behavior stigma items between and within groups. In bivariate analyses, we used log-binomial regression models to generate crude prevalence ratios (PRs) including 95% confidence intervals, which were used to test variables of interest for associations with stigma. Specifically, we examined the association of race/ethnicity, US region, population density, and age group with each sexual behavior stigma item in AMIS-2015. Within US/Africa regions, we measured the association between disclosure and stigma from family members and health care workers.

Multivariable log-binomial models were used to test for associations between region (United States, West Africa, Southern Africa) and prevalence of each stigma item after adjusting for potential confounders (age, disclosure, population density, and education level), which were identified based on findings from previous studies [16,39,43,51,52]. In addition, we accounted for nesting of participants within countries using a random intercept in each of the multivariable models, which is a method that has been used in similar studies [20,53,54]. Listwise deletion was used to handle missing data. Significance was determined at alpha = .05.

## Results

### Sample Demographics

In AMIS-2015, the median age of participants was 32 years (interquartile range (IQR)=24-50) (Table 2). The sample was roughly evenly divided across regions of the United States, with 19.9% (516/2589) from the Midwest, 18.3% (474/2589) from the Northeast, 37.1% (961/2589) from the South, and 24.6% (638/2589) from the West. Most lived in an urban (1749/2551, 68.6%) area. The distribution of race/ethnicity was as follows: 13.4% (342/2551) Hispanic, 7.4% (188/2551) non-Hispanic Black, 71.7% (1829/2551) non-Hispanic White, and 7.5% (192/2551) Other. The majority (1368/2569, 53.3%) had completed a college level education or higher, 35.0% (898/2569) completed some college, and 11.8% (303/2569) completed high school or lower. Most participants had disclosed their same-sex practices to either a family member (1982/2412, 82.2%) or health care worker (1735/2397, 72.4%).

In West Africa, participants were recruited from Nigeria (1067/3379, 31.6%), Cote d’Ivoire (794/3379, 23.5%), Togo (553/3379, 16.4%), Senegal (522/3379, 15.5%), and Burkina Faso (443/3379, 13.1%). The median age was 23 years (IQR=21-27). Most had completed at least a secondary school education (1898/3359, 56.5%), whereas 24.8% (834/3359) completed more than a secondary school education and 18.7% (627/3359) completed primary school or lower. Overall, 19.7% (665/3379) had disclosed their same-sex practices to a family member and 35.6% (1199/3379) had disclosed to a health care worker.

In Southern Africa, participants were recruited from Lesotho (487/906, 53.8%) and Swaziland (419/906, 46.3%). The median age was 24 years (IQR=21-28). Similar to West Africa, most (597/905, 66.0%) had completed a secondary school education, whereas 20.8% (188/905) completed more than a secondary school education, and 13.3% (120/905) completed primary school or less. Just less than one-third (277/901, 30.7%) disclosed same-sex practices to a family member and 12.2% (110/901) disclosed to a health care worker.

**Table 2:** Sample demographics by United States/Africa region.

		United States	Southern Africa	West Africa
		N (%)	N (%)	N (%)
**Median age (IQR)**		32 (24-50)	24 (21-28)	23 (21-27)
**Education completed**				
	High school/secondary school or less	303 (11.8)	717 (79.2)	2525 (75.2)
	More than secondary/high school	2266 (88.2)	188 (20.8)	834 (24.8)
**Population density**				
	Rural	802 (31.4)	603 (66.6)	597 (17.7)
	Urban	1749 (68.6)	303 (33.4)	2782 (82.3)
**Disclosed same-sex practices to health care worker**		1735 (72.4)	110 (12.2)	1199 (35.6)
**Disclosed same-sex practices to family member**		1982 (82.2)	277 (30.7)	665 (19.7)

### Sexual Behavior Stigma in AMIS-2015

**Figure 1:**
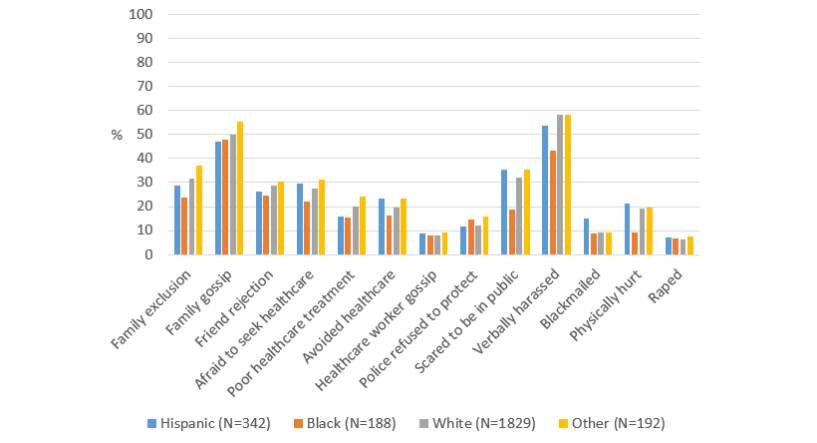
Prevalence of sexual behavior stigma among MSM in AMIS-2015 by race/ethnicity.

Overall prevalence of stigma items in AMIS-2015 are presented in Table 3. Briefly, the most commonly reported items were verbal harassment (1423/2509, 56.7%), family gossip (1155/2312, 50.0%), and being scared to be in public (811/2551, 31.8%). Prevalence of sexual behavior stigma by race/ethnicity within the United States are shown in Figure 1 and Multimedia Appendix 1. In the bivariate analyses, Hispanic MSM were more likely to report being blackmailed as compared with White MSM (*P*=.002). Black MSM were less likely than White MSM to report family exclusion (*P*=.04), fear of being in public (*P*<.001), verbal harassment (*P*<.001), and physical attacks (*P*=.002).

**Figure 2:**
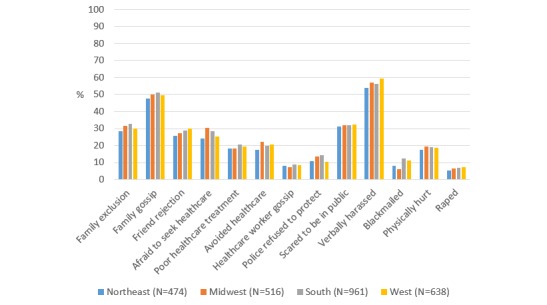
Prevalence of sexual behavior stigma among MSM in AMIS-2015 by United States region.

**Figure 3:**
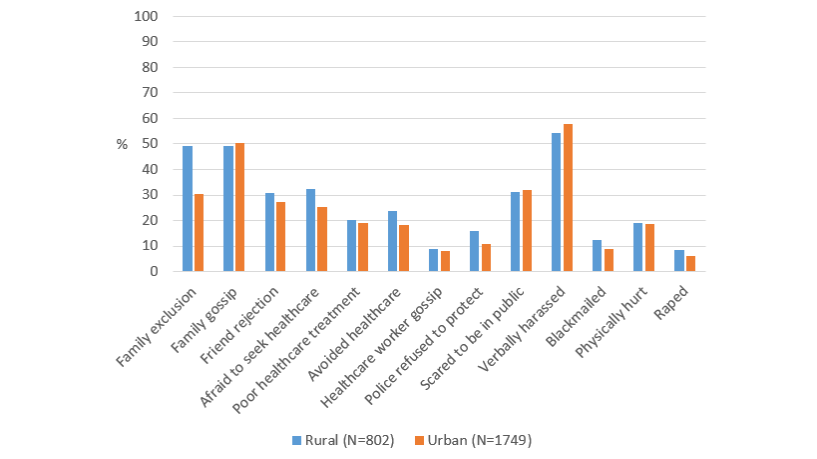
Prevalence of sexual behavior stigma among MSM in AMIS-2015 by population density.

There were no differences in sexual behavior stigma prevalence by United States region, except that MSM in the West were less likely to feel like police refused to protect them as compared with MSM in the South (*P*=.04), and MSM in the Midwest (*P*<.001), and Northeast (*P*=.02) were less likely to report blackmail as compared with MSM in the South (Figure 2 and Multimedia Appendix 2). MSM who lived in rural as compared with urban areas were more likely to report being afraid to seek health care (*P*<.001), avoiding health care (*P*=.003), feeling like police refused to protect (*P*=.001), being blackmailed (*P*=.004), and being raped (*P*=.04) (Figure 3 and Multimedia Appendix 3).

**Figure 4:**
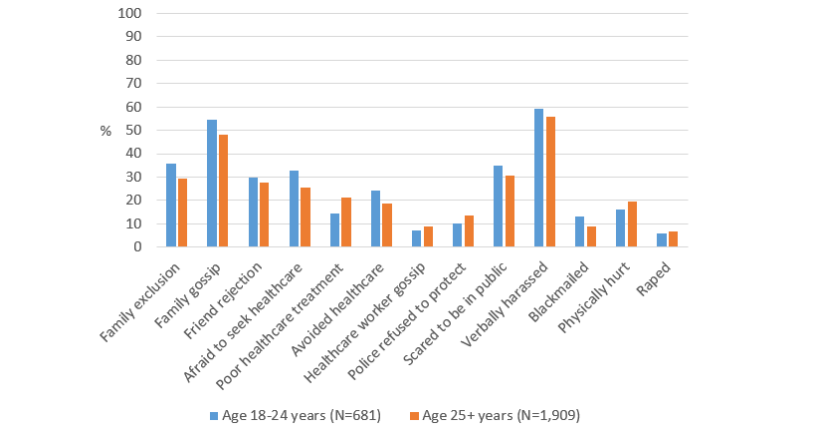
Prevalence of sexual behavior stigma among MSM in AMIS-2015 by age group.

Finally, young MSM (aged 18-24 years) were more likely than older MSM to be excluded by family members (*P*=.002), gossiped about by family members (*P*=.007), afraid to seek health care (*P*<.001), avoid seeking health care (*P*=.002), scared to be seen in public (*P*=.03), and to be blackmailed (*P*=.003) (Figure 4 and Multimedia Appendix 4). However, older MSM (aged 25 years and older) were more likely to have been treated poorly in a health care center (*P*<.001), and to feel like police refused to protect (*P*=.03).

### Comparison of Stigma Prevalence Across United States and Sub-Saharan Africa

**Figure 5:**
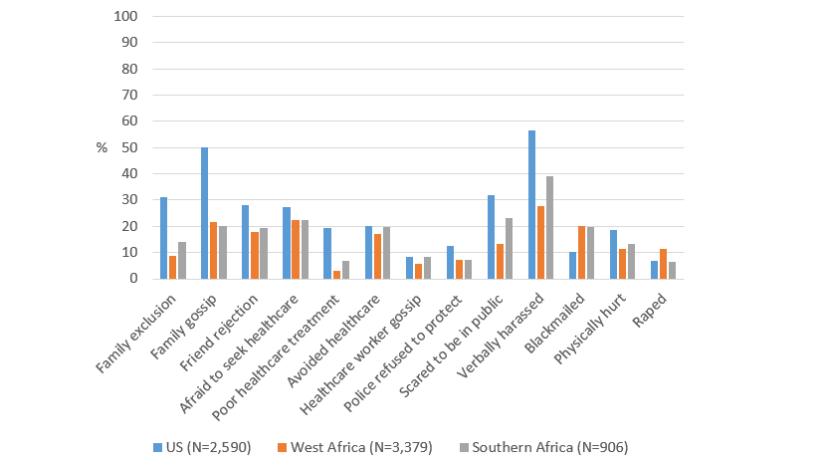
Prevalence of sexual behavior stigma among MSM by United States/Africa region.

Overall, sexual behavior stigma reported in AMIS-2015 was similar to or higher than what was reported by participants in the Sub-Saharan African studies (Figure 5 and Table 3). Family exclusion, family gossip, friend rejection, being afraid to seek health care, being treated poorly by a health care worker, feeling like police refused to protect, being scared to be in public, verbal harassment, and being physically hurt were all more commonly reported by AMIS-2015 participants (*P*<.001 for all, except *P*=.005 for comparing fear of seeking health care with Southern Africa). AMIS-2015 participants were also more likely than MSM in West Africa to report avoiding seeking health care (*P*=.002) and health care worker gossip (*P*<.001). Participants in West and Southern Africa more commonly reported blackmail (*P*<.001 for both) compared with those in the United States, and participants in West Africa more commonly reported rape as compared with MSM in the United States (*P*<.001). In all settings, MSM reported verbal harassment as the most common experience of stigma. Rape was the least common experience in United States and Southern Africa, whereas being treated poorly in a health care center was least commonly reported in West Africa.

**Table 3:** Prevalence of sexual behavior stigma among MSM by United States/Africa region.

Stigma	Region	n/N (%)	Prevalence ratio (95% confidence interval)	*P* value
**Family exclusion**	United States	775/2489 (31.1)	Reference	--
	Southern Africa	126/905 (13.9)	0.45 (0.38-0.53)	<.001
	West Africa	298/3378 (8.8)	0.28 (0.25-0.32)	<.001
**Family gossip**	United States	1155/2312 (50.0)	Reference	--
	Southern Africa	181/903 (20.0)	0.40 (0.35-0.46)	<.001
	West Africa	727/3377 (21.5)	0.43 (0.40-0.47)	<.001
**Friend rejection**	United States	677/2400 (28.2)	Reference	--
	Southern Africa	173/902 (19.2)	0.68 (0.59-0.79)	<.001
	West Africa	604/3378 (17.9)	0.63 (0.58-0.70)	<.001
**Afraid to seek health care**	United States	665/2429 (27.4)	Reference	--
	Southern Africa	203/905 (22.4)	0.82 (0.71-0.94)	.005
	West Africa	756/3378 (22.4)	0.82 (0.75-0.89)	<.001
**Poor healthcare treatment**	United States	462/2374 (19.5)	Reference	--
	Southern Africa	62/905 (6.9)	0.35 (0.27-0.45)	<.001
	West Africa	102/3355 (3.0)	0.16 (0.13-0.19)	<.001
**Avoided health care**	United States	488/2430 (20.1)	Reference	--
	Southern Africa	180/905 (19.9)	0.99 (0.85-1.15)	.90
	West Africa	573/3377 (17.0)	0.84 (0.76-0.94)	.002
**Health care worker gossip**	United States	201/2397 (8.4)	Reference	--
	Southern Africa	74/905 (8.2)	0.98 (0.76-1.26)	.85
	West Africa	195/3350 (5.8)	0.69 (0.57-0.84)	<.001
**Police refused to protect**	United States	298/2367 (12.6)	Reference	--
	Southern Africa	65/902 (7.2)	0.57 (0.44-0.74)	<.001
	West Africa	244/3374 (7.2)	0.57 (0.49-0.67)	<.001
**Scared to be in public**	United States	811/2551 (31.8)	Reference	--
	Southern Africa	208/905 (23.0)	0.72 (0.63-0.83)	<.001
	West Africa	452/3376 (13.4)	0.42 (0.38-0.47)	<.001
**Verbally harassed**	United States	1423/2509 (56.7)	Reference	--
	Southern Africa	351/905 (38.8)	0.68 (0.63-0.75)	<.001
	West Africa	934/3377 (27.7)	0.49 (0.46-0.52)	<.001
**Blackmailed**	United States	252/2507 (10.1)	Reference	--
	Southern Africa	178/905 (19.7)	1.96 (1.64-2.33)	<.001
	West Africa	686/3378 (20.3)	2.02 (1.77-2.31)	<.001
**Physically hurt**	United States	473/2513 (18.8)	Reference	--
	Southern Africa	120/903 (13.3)	0.71 (0.59-0.85)	<.001
	West Africa	388/3356 (11.6)	0.61 (0.54-0.70)	<.001
**Raped**	United States	159/2377 (6.7)	Reference	--
	Southern Africa	59/902 (6.5)	0.98 (0.73-1.31)	.88
	West Africa	388/3365 (11.5)	1.72 (1.44-2.06)	<.001

**Figure 6:**
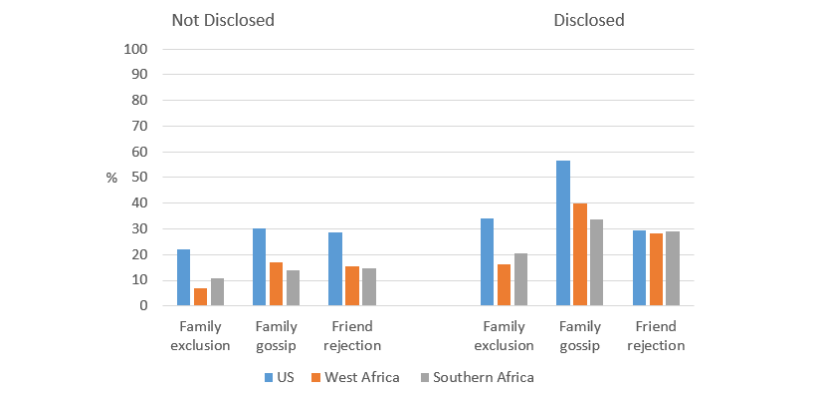
Prevalence of sexual behavior stigma among MSM who disclosed same-sex behaviors to family vs not disclosed.

Sexual behavior stigma from family and friends was higher among those who had disclosed their sexual practices to a family member in all regions (*P*<.001 for all); however, the prevalence of friend rejection among AMIS-2015 participants was similar between the two disclosure groups (*P*=.75) (Figure 6 and Multimedia Appendix 5).

**Figure 7:**
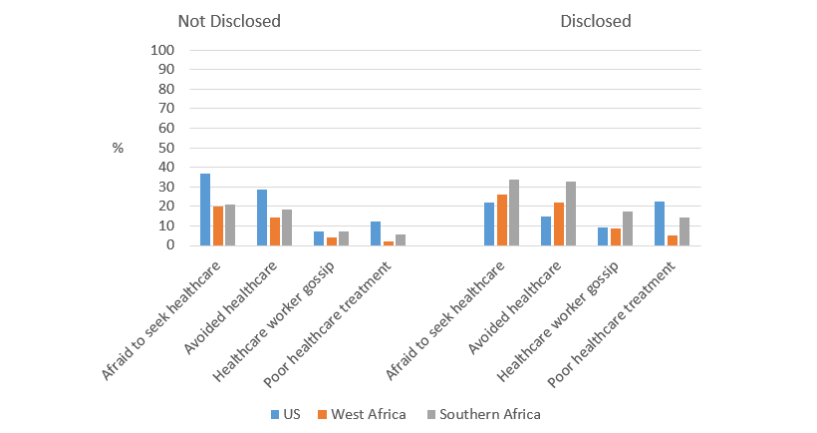
Prevalence of sexual behavior stigma among MSM who disclosed same-sex behaviors to health care worker vs not disclosed.

In the United States, participants who had disclosed their sexual practices to a health care worker were less likely to be afraid to seek health care (*P*<.001) or to avoid seeking health care (*P*<.001) as compared with those who had not disclosed; however, they were more likely than those who had not disclosed to report being treated poorly in a health care center (*P*<.001) (Figure 7 and Multimedia Appendix 6). In both West and Southern Africa, the prevalence of each health care–related sexual behavior stigma item increased among those who had disclosed versus not disclosed (*P*<.001 for all).

### Multivariable Adjusted Associations of United States/Africa Region With Sexual Behavior Stigma Items

After adjusting for age, disclosure status with family members and health care workers, education level, and population density, we found that AMIS-2015 participants continued to report higher levels of family exclusion (*P*=.02) and poor health care treatment (*P*=.009) as compared with West Africa (Table 4). However, participants in both West Africa (*P*<.001) and Southern Africa (*P*<.001) reported a higher prevalence of blackmail. The prevalence of all other types of stigma was not found to be statistically significantly different across settings.

**Table 4:** Adjusted associations of United States/Africa region with sexual behavior stigma.

Stigma	Region	Adjusted prevalence ratio^a^ (95% CI)	*P* value
**Family exclusion**	United States	Reference	--
	Southern Africa	0.52 (0.19-1.44)	.21
	West Africa	0.33 (0.13-0.82)	.02
**Family gossip**	United States	Reference	--
	Southern Africa	0.52 (0.20-1.35)	.18
	West Africa	0.50 (0.22-1.17)	.11
**Friend rejection**	United States	Reference	--
	Southern Africa	0.91 (0.42-1.97)	.81
	West Africa	0.75 (0.37-1.49)	.41
**Afraid to seek health care**	United States	Reference	--
	Southern Africa	0.79 (0.20-3.18)	.74
	West Africa	0.77 (0.22-2.67)	.68
**Poor health care treatment**	United States	Reference	--
	Southern Africa	0.52 (0.14-1.95)	.33
	West Africa	0.21 (0.06-0.67)	.009
**Avoided health care**	United States	Reference	--
	Southern Africa	0.91 (0.20-4.17)	.09
	West Africa	0.85 (0.22-3.32)	.82
**Health care worker gossip**	United States	Reference	--
	Southern Africa	1.25 (0.32-4.96)	.75
	West Africa	0.97 (0.29-3.30)	.97
**Police refused to protect**	United States	Reference	--
	Southern Africa	0.84 (0.08-8.38)	.88
	West Africa	0.59 (0.08-4.59)	.61
**Scared to be in public**	United States	Reference	--
	Southern Africa	0.71 (0.15-3.28)	.66
	West Africa	0.37 (0.09-1.45)	.15
**Verbally harassed**	United States	Reference	--
	Southern Africa	0.84 (0.44-1.59)	.59
	West Africa	0.52 (0.30-0.93)	.03
**Blackmailed**	United States	Reference	--
	Southern Africa	2.94 (1.70-5.07)	<.001
	West Africa	2.86 (1.76-4.64)	<.001
**Physically hurt**	United States	Reference	--
	Southern Africa	0.83 (0.17-4.07)	.92
	West Africa	0.56 (0.14-2.31)	.43
**Raped**	United States	Reference	--
	Southern Africa	0.94 (0.17-5.15)	.94
	West Africa	1.68 (0.37-7.62)	.50

^a^Models adjust for age, disclosure of same-sex behaviors to family or health care workers, education level, and population density. Models include a random intercept to account for nesting of participants within countries.

## Discussion

### Principal Results

In these analyses, we identified a high prevalence of experienced, anticipated, and perceived sexual behavior stigma for MSM in all settings. Based on Figure 5, the overall pattern of responses appeared to be similar across regions, with MSM in all settings most commonly reporting verbal harassment and less commonly reporting rape or being treated poorly in a health care center. Surprisingly, in many cases US participants reported even greater levels of stigma than MSM in settings where same sex practices were criminalized. However, many of these differences were not significant after adjusting for potential confounders, and may also be the result of inherent differences in sampling methodology (eg, Web-based questionnaire vs face-to-face interviews).

### Interpretation and Comparison with Prior Work

Traumatic experiences among MSM are problematic for the HIV response because they can lead to reduced uptake and use of health services [18,20,21,23,55]. Indeed, stigma has been associated with reduced rates of HIV testing, increased risk for HIV infection, increased fear and avoidance of health care, increased condomless anal sex, and reduced engagement in HIV treatment for those living with HIV [11-14,23]. Stigma has further been linked to adverse mental health outcomes such as reduced self-esteem, internalized homophobia, and depression, with potential mediation by resiliency and coping ability [56-59]. Thus, the findings of high prevalence of multiple forms of stigma suggest the potential for stigma mitigation interventions to improve existing mental health services and HIV prevention and treatment interventions for MSM.

Within AMIS-2015, there were minimal differences in report of sexual behavior stigma by race/ethnicity and by region, suggesting the pervasiveness of these experiences or perceptions among MSM in the United States. However, MSM in rural areas were more likely to report fear and avoidance of health care, feeling like police refused to protect, being blackmailed, and being raped. This is consistent with the findings from previous studies suggesting that stigma toward accessing mental health and HIV services is higher in rural regions of both the United States and Sub-Saharan Africa as compared with more urban regions [51,60,61]. Our finding that Black MSM experienced lower levels of certain types of sexual behavior stigma is in contrast to previous literature suggesting that stigma surrounding sexual orientation is more pervasive in the Black community [62,63]. However, much of this previous work assessed internalized homo-negativity among Black MSM, which we did not measure in this study [64]. In addition, there is potential selection bias in that Black MSM are underrepresented in AMIS-2015, and thus this finding should be further explored and confirmed using alternative sampling methods. There were some differences within AMIS-2015 between age groups, with young MSM (aged 18-24 years) being more likely than older MSM to report stigma from family, to be afraid to seek health care or avoid seeking health care, to be scared to be seen in public, and to be blackmailed. This is somewhat surprising given that we measured lifetime exposure to stigma and suspected that older MSM would have had more opportunity for exposure, and thus higher levels of stigma. These high levels of anticipated stigma and particularly fear and avoidance of health care among young MSM are important to address, as they likely contribute to the sustained or growing incidence of HIV among young MSM worldwide [65-69].

When we stratified by disclosure status, the prevalence of stigma from family members increased for participants who had disclosed same-sex practices to family as compared with those who had not disclosed, and this occurred within each setting of the United States, West Africa, and Southern Africa. This is likely reflective of the fact that in all of these settings MSM who have disclosed their same sex practices are more easily identified as targets for stigma, discrimination, and harassment [43,70-72]. However, the patterns of stigma associated with disclosure status were somewhat different when we examined health care–related stigma. In the United States, those who had disclosed same-sex practices to a health care worker were less likely to fear or avoid seeking health care services, although they were more likely to report being treated poorly in a health care center. Based on these findings, it seems possible that MSM who have disclosed to their providers are more comfortable seeking health care, even though it can sometimes result in negative experiences. In Sub-Saharan Africa, health care stigma increased among those who had disclosed versus not disclosed, indicating the immediate need for structural interventions to improve access to culturally competent health care for MSM across all settings [1,22,73].

There have been successful efforts in Sub-Saharan Africa and other regions to increase clinical and cultural competency for health care workers who provide HIV and sexually transmitted infection prevention, treatment, and care to MSM patients [74-78]. Given our findings, these efforts should be intensified and expanded to cover all domains of health care, particularly because the proper training of health care professionals tends to be one of the more easily implementable intervention strategies to reduce or mitigate stigma. However, stigma toward MSM is not limited to health care settings and structural interventions may also be needed to reduce stigma in community settings including schools, workplaces, churches, and families. These interventions would need to be appropriately tailored to meet the needs of different cultures and communities. In the United States, for example, gay-straight alliances have been successful in reducing homophobia in schools, although this method may or may not be suitable for African settings [79]. Eventual acceptance of lesbian, gay, bisexual, and transgender individuals may be inevitable with slowly increasing social acceptance over time; however, these social changes are not happening at the pace required to make an immediate impact on reducing negative health outcomes.

### Limitations

Although we restricted the study populations from each data set to be as similar as possible (eg, aged 18 years and older, anal sex with a man in the past 12 months, cis-gender male) and performed adjusted analyses, there are some inherent differences between the United States and Sub-Saharan Africa study populations that cannot be adjusted for, including sampling strategies, mode of survey administration, and time period of study participation. Because AMIS-2015 was an anonymous Web-based survey, it is possible some participants were more comfortable to disclose this sensitive information in an anonymous Internet setting as compared to during a face-to-face interview [80,81]. This bias might be mitigated because interviewers were administered by highly trained MSM or MSM-friendly staff members. Moreover, participants in these studies reported high levels of another sensitive issue, condomless anal sex, which further suggests minimal bias due to social desirability (data not shown).

Another limitation is that the African studies were conducted with incentives for participation that were not included in AMIS, including in some cases HIV and biological testing, and therefore may have reached individuals who would have not otherwise participated. Participants in AMIS were highly educated and mostly of White, non-Hispanic race/ethnicity, suggesting limitations to generalizability to other MSM in the United States. It is also possible that AMIS participants, who were recruited from gay-related websites, were more actively involved in the gay community and more likely to be exposed to stigma. This is because participants in Sub-Saharan Africa were recruited using RDS (except in Swaziland), which has been shown to be capable of reaching MSM who are less engaged in the gay community and HIV testing [82]. Finally, the prevalence of missing values for AMIS-2015 (1.5%-10.7% for each stigma item) were higher than those in Western and Southern Africa (0%-1%), likely because AMIS-2015 was a Web-based survey, whereas African data were collected during face-to-face interviews. However, this study is the first to our knowledge to make a direct comparison of sexual behavior stigma between MSM in the United States and Sub-Saharan Africa using consistent metrics.

### Conclusions

Sexual behavior stigma felt or experienced by MSM appears to be pervasive across the United States and Sub-Saharan Africa, and this has implications for both sexual and mental health. Given the growing desire to measure, quantify, and evaluate stigma toward key populations as part of the HIV response, this paper is timely in its comparison of prevalence of sexual behavior stigma across 3 widely different regions (the United States and Western and Southern Sub-Saharan Africa). It is important to note that results may be influenced by differences in sampling methodology across regions. However, the consistently observed high burden of stigma points to the need for immediate structural interventions to address each of the domains of sexual behavior stigma presented here; that is, stigma from family and friends, health care workers, and from broader society. These interventions will be critical for making a positive impact on the mental health of these men, and also for reducing the global sustained burden of HIV and other adverse health outcomes in this key population.

## Appendix

Multimedia Appendix 1 Prevalence of sexual behavior stigma among MSM in AMIS-2015 by race/ethnicity.

Multimedia Appendix 2 Prevalence of sexual behavior stigma among MSM in AMIS-2015 by United States region.

Multimedia Appendix 3 Prevalence of sexual behavior stigma among MSM in AMIS-2015 by population density.

Multimedia Appendix 4 Prevalence of sexual behavior stigma among MSM in AMIS-2015 by age group.

Multimedia Appendix 5 Prevalence of sexual behavior stigma among MSM who disclosed same-sex behaviors to family vs. not disclosed, by United States/Africa region.

Multimedia Appendix 6 Prevalence of sexual behavior stigma among MSM who disclosed same-sex behaviors to healthcare worker vs. not disclosed, by United States/Africa region.

## Abbreviations

CBOs: community-based organizations
CI: confidence interval
HIV: human immunodeficiency virus
IQR: interquartile range
IRB: institutional review board
MSM: men who have sex with men
PR: prevalence ratio
RDS: respondent driven sampling
