# Supplementary material for: The Prevalence of Sexual Behavior Stigma Affecting Gay Men and Other Men Who Have Sex with Men Across Sub-Saharan Africa and in the United States
Source: JMIR Public Health Surveill. 2016 Jul 26;2(2):e35. doi: 10.2196/publichealth.5824 (PMC4978863; doi:10.2196/publichealth.5824)
Supplement: Multimedia Appendix 1 [file publichealth_v2i2e35_app1.pdf]

Supplemental Table 1. Prevalence of sexual behavior stigma among MSM in AMIS-2015 by race/ethnicity

Stigma	Race/ethnicity	n/N (%)	PR (95% CI)	*P*-value
Family exclusion	Black	43/181 (23.8)	0.75 (0.57-0.98)	.04
	Hispanic	92/320 (28.8)	0.91 (0.75-1.09)	.30
	Other	67/181 (37.0)	1.17 (0.95-1.43)	.13
	White	562/1772 (31.7)	Reference	--
Family gossip	Black	76/159 (47.8)	0.96 (0.81-1.14)	.62
	Hispanic	144/305 (47.2)	0.95 (0.83-1.08)	.40
	Other	97/175 (55.4)	1.11 (0.96-1.28)	.14
	White	818/1640 (49.9)	Reference	--
Friend rejection	Black	41/166 (24.7)	0.86 (0.65-1.13)	.29
	Hispanic	83/314 (26.4)	0.92 (0.76-1.12)	.42
	Other	56/184 (30.4)	1.06 (0.84-1.34)	.62
	White	488/1701 (28.7)	Reference	--
Afraid to seek healthcare	Black	38/171 (22.2)	0.81 (0.61-1.09)	.17
	Hispanic	94/318 (29.6)	1.08 (0.90-1.30)	.40
	Other	58/185 (31.4)	1.15 (0.92-1.44)	.23
	White	469/1718 (27.3)	Reference	--
Poor healthcare treatment	Black	26/169 (15.4)	0.77 (0.54-1.11)	.17
	Hispanic	49/307 (16.0)	0.80 (0.61-1.05)	.11
	Other	43/179 (24.0)	1.21 (0.91-1.59)	.18
	White	335/1683 (19.9)	Reference	--
Avoided healthcare	Black	28/172 (16.3)	0.83 (0.58-1.17)	.29
	Hispanic	74/317 (23.3)	1.18 (0.95-1.48)	.13
	Other	43/185 (23.2)	1.18 (0.89-1.56)	.25
	White	339/1720 (19.7)	Reference	--
Healthcare worker gossip	Black	14/172 (8.1)	1.00 (0.59-1.70)	.99
	Hispanic	27/312 (8.7)	1.07 (0.72-1.58)	.75
	Other	17/182 (9.3)	1.15 (0.71-1.86)	.57
	White	138/1699 (8.1)	Reference	--
Police refused to protect	Black	25/171 (14.6)	1.21 (0.83-1.78)	.32
	Hispanic	36/306 (11.8)	0.98 (0.70-1.36)	.89
	Other	28/178 (15.7)	1.31 (0.91-1.88)	.15
	White	202/1678 (12.0)	Reference	--
Scared to be in public	Black	35/186 (18.8)	0.58 (0.43-0.79)	<.001
	Hispanic	119/336 (35.4)	1.10 (0.94-1.29)	.24
	Other	66/186 (35.5)	1.10 (0.90-1.35)	.36
	White	582/1806 (32.2)	Reference	--
Verbally harassed	Black	77/178 (43.3)	0.74 (0.62-0.88)	<.001
	Hispanic	178/331 (53.8)	0.92 (0.83-1.03)	.14
	Other	108/185 (58.4)	1.00 (0.88-1.14)	.99
	White	1037/1778 (58.3)	Reference	--
Blackmailed	Black	15/175 (8.6)	0.92 (0.55-1.52)	.74
	Hispanic	49/330 (14.9)	1.59 (1.18-2.14)	.002
	Other	17/187 (9.1)	0.97 (0.60-1.57)	.91
	White	166/1777 (9.3)	Reference	--
Physically hurt	Black	16/176 (9.1)	0.47 (0.29-0.76)	.002
	Hispanic	70/332 (21.1)	1.10 (0.87-1.38)	.43
	Other	36/184 (19.6)	1.02 (.75-1.38)	.91
	White	343/1783 (19.2)	Reference	--
Raped	Black	11/168 (6.6)	1.01 (0.55-1.83)	.98
	Hispanic	22/310 (7.1)	1.09 (.70-1.70)	.70
	Other	13/174 (7.5)	1.15 (0.66-2.00)	.62
	White	110/1693 (6.5)	Reference	--
