# Supplementary material for: The Prevalence of Sexual Behavior Stigma Affecting Gay Men and Other Men Who Have Sex with Men Across Sub-Saharan Africa and in the United States
Source: JMIR Public Health Surveill. 2016 Jul 26;2(2):e35. doi: 10.2196/publichealth.5824 (PMC4978863; doi:10.2196/publichealth.5824)
Supplement: Multimedia Appendix 2 [file publichealth_v2i2e35_app2.pdf]

Supplemental Table 2. Prevalence of sexual behavior stigma among MSM in AMIS-2015 by United States region

Stigma	US region	n/N (%)	PR (95% CI)	*P*-value
Family exclusion	Northeast	129/454 (28.4)	0.87 (0.73-1.03)	.10
	Midwest	158/501 (31.5)	0.96 (0.82-1.13)	.63
	West	184/609 (30.2)	0.92 (0.79-1.07)	.29
	South	303/924 (32.8)	Reference	--
Family gossip	Northeast	201/421 (47.7)	0.94 (0.83-1.06)	.28
	Midwest	235/469 (50.1)	0.98 (0.88-1.10)	.76
	West	287/576 (49.8)	0.97 (0.88-1.09)	.66
	South	431/845 (51.0)	Reference	--
Friend rejection	Northeast	113/440 (25.7)	0.89 (0.74-1.08)	.23
	Midwest	132/485 (27.2)	0.94 (0.79-1.13)	.52
	West	179/597 (30.0)	1.04 (0.89-1.22)	.64
	South	253/877 (28.9)	Reference	--
Afraid to seek healthcare	Northeast	107/446 (24.0)	0.84 (0.69-1.02)	.08
	Midwest	149/490 (30.4)	1.06 (0.90-1.26)	.47
	West	153/599 (25.5)	0.89 (0.75-1.06)	.20
	South	255/893 (28.6)	Reference	--
Poor healthcare treatment	Northeast	81/440 (18.4)	0.89 (0.71-1.13)	.35
	Midwest	87/477 (18.2)	0.88 (0.70-1.11)	.30
	West	113/578 (19.6)	0.95 (0.77-1.17)	.62
	South	181/878 (20.6)	Reference	--
Avoided healthcare	Northeast	78/448 (17.4)	0.87 (0.69-1.11)	.27
	Midwest	109/489 (22.3)	1.12 (0.91-1.38)	.30
	West	123/599 (20.5)	1.03 (0.84-1.26)	.78
	South	178/893 (19.9)	Reference	--
Healthcare worker gossip	Northeast	36/440 (8.2)	0.91 (0.62-1.32)	.61
	Midwest	34/474 (7.2)	0.79 (0.54-1.17)	.24
	West	51/596 (8.6)	0.95 (0.68-1.33)	.75
	South	80/886 (9.0)	Reference	--
Police refused to protect	Northeast	47/438 (10.7)	0.75 (0.55-1.03)	.07
	Midwest	65/474 (13.7)	0.96 (0.73-1.27)	.77
	West	62/587 (10.6)	0.74 (0.55-0.98)	.04
	South	124/867 (14.3)	Reference	--
Scared to be in public	Northeast	146/467 (31.3)	0.98 (0.84-1.16)	.85
	Midwest	162/507 (32.0)	1.01 (0.86-1.18)	.94
	West	203/631 (32.2)	1.01 (0.88-1.17)	.86
	South	300/945 (31.8)	Reference	--
Verbally harassed	Northeast	245/455 (53.9)	0.96 (0.86-1.06)	.39
	Midwest	285/501 (56.9)	1.01 (0.92-1.11)	.84
	West	367/618 (59.4)	1.05 (0.97-1.15)	.23
	South	526/934 (56.3)	Reference	--
Blackmailed	Northeast	37/459 (8.1)	0.66 (0.46-0.94)	.02
	Midwest	31/499 (6.2)	0.51 (0.35-0.74)	<.001
	West	69/617 (11.2)	0.91 (0.69-1.21)	.53
	South	114/931 (12.2)	Reference	--
Physically hurt	Northeast	79/458 (17.3)	0.90 (0.71-1.15)	.41
	Midwest	99/504 (19.6)	1.03 (0.82-1.28)	.80
	West	116/618 (18.8)	0.98 (0.80-1.21)	.87
	South	178/932 (19.1)	Reference	--
Raped	Northeast	24/438 (5.5)	0.78 (0.49-1.23)	.29
	Midwest	31/475 (6.5)	0.93 (0.61-1.41)	.73
	West	42/579 (7.3)	1.03 (0.71-1.51)	.86
	South	62/884 (7.0)	Reference	--
