# Supplementary material for: The Prevalence of Sexual Behavior Stigma Affecting Gay Men and Other Men Who Have Sex with Men Across Sub-Saharan Africa and in the United States
Source: JMIR Public Health Surveill. 2016 Jul 26;2(2):e35. doi: 10.2196/publichealth.5824 (PMC4978863; doi:10.2196/publichealth.5824)
Supplement: Multimedia Appendix 3 [file publichealth_v2i2e35_app3.pdf]

Supplemental Table 3. Prevalence of sexual behavior stigma among MSM in AMIS-2015 by population density

Stigma	Population Density	n/N (%)	PR (95% CI)	*P*-value
Family exclusion	Rural	352/717 (49.1)	1.08 (0.95-1.22)	.25
	Urban	512/1680 (30.5)	Reference	--
Family gossip	Rural	352/717 (49.1)	0.98 (0.89-1.07)	.62
	Urban	783/1559 (50.2)	Reference	--
Friend rejection	Rural	226/739 (30.6)	1.12 (0.98-1.29)	.09
	Urban	442/1625 (27.2)	Reference	--
Afraid to seek healthcare	Rural	241/749 (32.2)	1.27 (1.12-1.46)	<.001
	Urban	415/1644 (25.2)	Reference	--
Poor healthcare treatment	Rural	150/735 (20.4)	1.08 (0.90-1.28)	.44
	Urban	305/1602 (19.0)	Reference	--
Avoided healthcare	Rural	177/749 (23.6)	1.28 (1.09-1.51)	.003
	Urban	303/1644 (18.4)	Reference	--
Healthcare worker gossip	Rural	66/736 (9.0)	1.12 (0.84-1.49)	.43
	Urban	130/1625 (8.0)	Reference	--
Police refused to protect	Rural	116/735 (15.8)	1.43 (1.15-1.78)	.001
	Urban	176/1596 (11.0)	Reference	--
Scared to be in public	Rural	246/791 (31.1)	0.97 (0.86-1.10)	.61
	Urban	553/1722 (32.1)	Reference	--
Verbally harassed	Rural	424/778 (54.5)	0.94 (0.87-1.02)	.13
	Urban	978/1692 (57.8)	Reference	--
Blackmailed	Rural	97/777 (12.5)	1.43 (1.12-1.82)	.004
	Urban	148/1692 (8.8)	Reference	--
Physically hurt	Rural	148/780 (19.0)	1.01 (0.85-1.21)	.87
	Urban	317/1695 (18.7)	Reference	--
Raped	Rural	61/737 (8.3)	1.38 (1.02-1.88)	.04
	Urban	96/1605 (6.0)	Reference	--
