# Supplementary material for: The Prevalence of Sexual Behavior Stigma Affecting Gay Men and Other Men Who Have Sex with Men Across Sub-Saharan Africa and in the United States
Source: JMIR Public Health Surveill. 2016 Jul 26;2(2):e35. doi: 10.2196/publichealth.5824 (PMC4978863; doi:10.2196/publichealth.5824)
Supplement: Multimedia Appendix 4 [file publichealth_v2i2e35_app4.pdf]

Supplemental Table 4. Prevalence of sexual behavior stigma among MSM in AMIS-2015 by age group

Stigma	Age group	n/N (%)	PR (95% CI)	*P*-value
Family exclusion	18-24 years	232/648 (35.8)	1.21 (1.07-1.38)	.002
	25+ years	543/1841 (29.5)	Reference	--
Family gossip	18-24 years	332/609 (54.5)	1.13 (1.03-1.23)	.007
	25+ years	823/1703 (48.3)	Reference	--
Friend rejection	18-24 years	188/634 (29.7)	1.07 (0.93-1.23)	.34
	25+ years	489/1766 (27.7)	Reference	--
Afraid to seek healthcare	18-24 years	207/635 (32.6)	1.28 (1.11-1.46)	<.001
	25+ years	458/1794 (25.5)	Reference	--
Poor healthcare treatment	18-24 years	91/622 (14.6)	0.69 (0.56-0.85)	<.001
	25+ years	371/1752 (21.2)	Reference	--
Avoided healthcare	18-24 years	155/641 (24.2)	1.30 (1.10-1.54)	.002
	25+ years	333/1789 (18.6)	Reference	--
Healthcare worker gossip	18-24 years	45/631 (7.1)	0.81 (0.59-1.11)	.19
	25+ years	156/1766 (8.8)	Reference	--
Police refused to protect	18-24 years	63/623 (10.1)	0.75 (0.58-0.98)	.03
	25+ years	235/1744 (13.5)	Reference	--
Scared to be in public	18-24 years	235/669 (35.1)	1.15 (1.01-1.30)	.03
	25+ years	576/1882 (30.6)	Reference	--
Verbally harassed	18-24 years	392/662 (59.2)	1.06 (0.98-1.14)	.12
	25+ years	1031/1847 (55.8)	Reference	--
Blackmailed	18-24 years	86/662 (13.0)	1.44 (1.13-1.84)	.003
	25+ years	166/1845 (9.0)	Reference	--
Physically hurt	18-24 years	107/659 (16.2)	0.82 (0.68-1.00)	.051
	25+ years	366/1854 (19.7)	Reference	--
Raped	18-24 years	37/619 (6.0)	0.86 (0.60-1.23)	.41
	25+ years	122/1758 (6.9)	Reference	--
