# Supplementary material for: The Prevalence of Sexual Behavior Stigma Affecting Gay Men and Other Men Who Have Sex with Men Across Sub-Saharan Africa and in the United States
Source: JMIR Public Health Surveill. 2016 Jul 26;2(2):e35. doi: 10.2196/publichealth.5824 (PMC4978863; doi:10.2196/publichealth.5824)
Supplement: Multimedia Appendix 5 [file publichealth_v2i2e35_app5.pdf]

Supplemental Table 5. Prevalence of sexual behavior stigma among MSM who disclosed same-sex behaviors to family vs. not disclosed, by United States/Africa region

Stigma	Region	Disclosed status	n/N (%)	PR (95% CI)	*P*-value
Family exclusion	US	Disclosed	655/1916 (34.2)	1.56 (1.29-1.90)	<.001
		Not Disclosed	89/407 (21.9)	Reference	--
	Southern Africa	Disclosed	57/277 (20.6)	1.89 (1.37-2.60)	<.001
		Not Disclosed	68/623 (10.9)	Reference	--
	West Africa	Disclosed	107/665 (16.1)	2.29 (1.83-2.85)	<.001
		Not Disclosed	191/2713 (7.0)	Reference	--
Family gossip	US	Disclosed	1011/1789 (56.5)	1.86 (1.59-2.18)	<.001
		Not Disclosed	115/379 (30.3)	Reference	--
	Southern Africa	Disclosed	93/276 (33.7)	2.41 (1.87-3.11)	<.001
		Not Disclosed	87/622 (14.0)	Reference	--
	West Africa	Disclosed	265/665 (39.9)	2.34 (2.06-2.65)	<.001
		Not Disclosed	462/2712 (17.0)	Reference	--
Friend rejection	US	Disclosed	547/1863 (29.4)	1.03 (0.87-1.22)	.75
		Not Disclosed	111/389 (28.5)	Reference	--
	Southern Africa	Disclosed	80/276 (29.0)	1.96 (1.50-2.55)	<.001
		Not Disclosed	92/621 (14.8)	Reference	--
	West Africa	Disclosed	189/665 (28.4)	1.86 (1.60-2.16)	<.001
		Not Disclosed	415/2713 (15.3)	Reference	--
