# Supplementary material for: The Prevalence of Sexual Behavior Stigma Affecting Gay Men and Other Men Who Have Sex with Men Across Sub-Saharan Africa and in the United States
Source: JMIR Public Health Surveill. 2016 Jul 26;2(2):e35. doi: 10.2196/publichealth.5824 (PMC4978863; doi:10.2196/publichealth.5824)
Supplement: Multimedia Appendix 6 [file publichealth_v2i2e35_app6.pdf]

Supplemental Table 6. Prevalence of sexual behavior stigma among MSM who disclosed same-sex behaviors to healthcare worker vs. not disclosed, by United States/Africa region

Stigma	Region	Disclosed status	n/N (%)	PR (95% CI)	*P*-value
Afraid to seek healthcare	US	Disclosed	364/1644 (22.1)	0.60 (0.52-0.69)	<.001
		Not Disclosed	228/619 (36.8)	Reference	--
	Southern Africa	Disclosed	37/110 (33.6)	1.60 (1.19-2.15)	<.001
		Not Disclosed	166/790 (21.0)	Reference	--
	West Africa	Disclosed	314/1199 (26.2)	1.30 (1.15-1.48)	<.001
		Not Disclosed	436/2167 (20.1)	Reference	--
Poor healthcare treatment	US	Disclosed	364/1621 (22.5)	1.80 (1.43-2.27)	<.001
		Not Disclosed	74/593 (12.5)	Reference	--
	Southern Africa	Disclosed	16/110 (14.6)	2.50 (1.47-4.26)	<.001
		Not Disclosed	46/790 (5.8)	Reference	--
	West Africa	Disclosed	61/1199 (5.1)	2.73 (1.84-4.04)	<.001
		Not Disclosed	40/2144 (1.9)	Reference	--
Avoided healthcare	US	Disclosed	247/1644 (15.0)	0.53 (0.44-0.62)	<.001
		Not Disclosed	177/621 (28.5)	Reference	--
	Southern Africa	Disclosed	36/110 (32.7)	1.80 (1.32-2.44)	<.001
		Not Disclosed	144/790 (18.2)	Reference	--
	West Africa	Disclosed	262/1199 (21.9)	1.55 (1.34-1.80)	<.001
		Not Disclosed	305/2166 (14.1)	Reference	--
Healthcare worker gossip	US	Disclosed	147/1624 (9.1)	1.23 (0.89-1.69)	.21
		Not Disclosed	45/611 (7.4)	Reference	--
	Southern Africa	Disclosed	19/110 (17.3)	2.48 (1.53-4.02)	<.001
		Not Disclosed	55/790 (7.0)	Reference	--
	West Africa	Disclosed	103/1198 (8.6)	2.04 (1.55-2.69)	<.001
		Not Disclosed	90/2140 (4.2)	Reference	--
